# Intimate partner violence, psychopathology and the women with schizophrenia in an outpatient clinic South-South, Nigeria

**DOI:** 10.1186/s12888-016-0898-2

**Published:** 2016-06-10

**Authors:** Taiwo Opekitan Afe, Thomas Chimezie Emedoh, Olawale Ogunsemi, Abosede Adekeji Adegbohun

**Affiliations:** Olabisi Onabanjo University, Ago Iwoye, Ogun Nigeria; Federal Neuro-psychiatric Hospital Calabar, Cross River, Calabar, Nigeria; Federal Neuro –Psychiatric Hospital, Lagos, Nigeria

**Keywords:** Intimate partner violence, Schizophrenia, Women, Psychopathology

## Abstract

**Background:**

Women with schizophrenia are a vulnerable risk group for intimate partner violence (1PV). There are few surveys that highlight the pattern, prevalence and association of IPV with psychopathology in these vulnerable group of women in South-South Nigeria. The aim of the study was to survey the forms, prevalence and association of Intimate partner violence with psychopathology.

**Method:**

The study was a cross-sectional survey of 77 female patients diagnosed with schizophrenia who were outpatients at the Federal Neuro-psychiatric Hospital, Calabar, Cross-River State in South-South region of Nigeria.

**Results:**

A total of 58 out of 77 (75 %) reported at least a form of IPV, Verbal abuse was the most prevalent form of IPV reported by participants (73 %, *n* = 56). Women who were younger were more likely to report verbal and sexual assault at *p* < 0.05. A shorter length of intimate relationship was significantly associated with sexual assault at *p* < 0.05. Sexual assault, verbal and physical abuse were significantly associated with higher mean score on the Brief Psychiatric Rating Scale at *p* = 0.01.

**Conclusion:**

The study highlighted the high rate of various forms of IPV among women with schizophrenia. Sexual assault, verbal and physical abuse were strongly associated with psychopathology. There is a need to identify risk of IPV among this vulnerable group by routine enquiry by clinicians’ and plan therapy accordingly. Holistic management is needed in management of victims in their care.

## Background

Intimate partner violence (IPV) has generated much public health concern [1, 2]. This is due to the increasing reports orchestrated by various news media globally. It has permeated every society and has become the most common form of gender based violence with resultant short and long term health consequences on victims [3, 4].

Intimate partner violence has been defined as any behaviour within an intimate relationship that causes physical, sexual or psychological harm [2]. The definition encompasses a broad range of physical aggression, sexual coercion, psychological abuse and controlling behaviours. This definition includes violence by both current and former partners.

Population based prevalence estimates do not usually give exact representation of the magnitude of the problem as most cases go unreported to the authorities [5]. Regardless of the limitation of underreporting, intimate partner violence constitutes significant crime statistics in every society. The lifetime prevalence of studies in different countries in 2005 ranged between 10–71 % from the World Health Multi-country Study [6]. In the United States, more than1 in 3 women and 1 in 4 men in the US have experienced rape, physical violence and/or stalking by a partner with IPV-related impact. IPV was implicated in 74 % of murders and suicides, 96 % of these were females. On average, 20 persons per minute are victims of partner violence [7, 8]. Unfortunately, in Nigeria there are no official statistics of such kind of violence due to underreporting and a cultural orientation that lends support to such acts against women [9, 10]. However, in a review of several pockets of studies in different parts of Nigeria, Olayanju et al. reported prevalence estimates ranging between 31–36 %, with women as the major victims [11]. Factors accounting for high risk of IPV among such women included younger age, low income, number of children and substance use by spouse. They also observed that as high as 45 % of those who experienced IPV involving physical and sexual forms fail to report [11].

Estimates of intimate partner violence among women with schizophrenia and its impact on the severity of the disorder are rare. Yet, this group of women constitute a vulnerable risk group for IPV when they get involved in intimate relationships. The chronic, debilitating, disorganising effect of the disease, the social stigmatisation and the overrepresentation among the low income groups are some of the inherent factors that make these women particularly vulnerable to abusive and violent intimate relationships [12, 13].

The consequences of intimate partner violence should be of major concern to mental health practitioners involved in the treatment of women with schizophrenia. The resultant effect can complicate health care treatments and quality of life of women with schizophrenia who are in such relationships [14]. Victims often bear a double burden as sufferers and victims. Unfortunately, these patients find it hard to disclose to their healthcare practitioners possibly due to lack of trust and further fear of victimisation by intimate partners [14]. Conversely, many mental health care practitioners fail to probe or show reticence to ask about intimate partner violence during routine consultation visits [15, 16].

Complicating the situation is the dearth of research and data available to mental health practitioners in Nigeria on the prevalence, types and the association with psychopathology in women with schizophrenia. Most often, enquires about IPV do not form part of routine questions at outpatient clinic visitations. Within this background the aim of this study was to survey and explore the various pattern of IPV associated with women with schizophrenia and the association with psychopathology.

## Methods

### Setting

The Federal Psychiatric Hospital Calabar is the first Neuro-Psychiatric Hospital in Nigeria. It is the only federally funded specialist psychiatric hospital in the south-south region of Nigeria. The Adult out-patient clinic attends to patients from 18 years and above. The clinics are supervised by consultant psychiatrists. The clinic operates on weekdays from Mondays to Fridays except Wednesdays. Consultation hours are between 8 am -4 pm. The clinic attends to patient on schedule appointments. The Hospital Records Department maintains a record of schedule appointments for each clinic day. Majority of the patients are from the south-south and south-east region of the Country.

### Study design

The study was a cross-sectional descriptive survey of women with schizophrenia. The participants were recruited from the attendees at the clinic. The period of study was from April 2014 to August2014.

### Participants

The respondents who participated in the study were chosen using the ballot method (with picks of YES or No) from the attendees at the clinics. The sampling frame included all adult female patients (≥18) who had been diagnosed with schizophrenia by a Consultant Psychiatrist and were on appointment. Those who were formally married or were currently living with a partner within the last one year were selected for the ballot process on each clinic day. The list of the attendees who met the criteria were obtained from direct enquiries from patients at the clinic before the start of the clinic. Patients with associated cognitive disorders or who were unable to sustain an interview process were excluded. The cognitive assessment was done clinically using the the Mini-mental State examination(cut-off of 25). In all Seventy-seven (77) respondents completed the survey out of the ninety-one persons (91) that were recruited (response rate 85 %). These participants were adjudged to be in stable condition for a one on one interview process. All the respondents gave their voluntary or written consent before the interview. The participants were adequately compensated with premium pass (express service delivery) within the hospital. Premium pass delivery is an express service delivery which reduces waiting times at the Drug collection centre and booking for next visit as the services were quicker as opposed to the regular service delivery in which waiting times are usually longer and services are not centralised within the same location. The premium parts takes about 30 min from the collection of drugs to booking of appointments. The regular pass takes more than 2 h from collection of drugs to booking of appointments because of the large number of patients and services are cheaper.

### Procedure

The interviews were conducted by female resident doctors who were trained in the use of the various instruments and conducting interviews with participants. Resident doctors that were recruited for the study were at least 2 years in Psychiatry residency training and were bilingual. They were fluent in English and Pidgin English (the local brand of English that was easily understood). The interview was done in a private office setting, on a one on one interview, strictly with the participants. The interviews were done after due consultations with their regular doctors, so the interviewers did not provide direct treatment to these patients. Participants were informed that the survey was about the status of their health and were assured on their anonymity. All participants inclusive of those who declined were assured that their treatment would not be affected in any way. An interview coordinator was appointed who was responsible for monitoring the interview process on each clinic day. Before the actual commencement of the study, a pilot study involving about 10 female patients was first conducted within the first month. This enabled us to reword and make better arrangements for the main study. The participants were well briefed as to the purpose of the interview.

### Instruments

Research Version of Structured Clinical Interview SCID. (SCID-1).

1. The Structured Clinical Interview for DSM-IV (SCID) was used to establish the diagnosis of schizophrenia among the participants. It assess psychiatric disorders described in the fourth (IV) edition of the] Diagnostic and Statistical Manual (DSM-IV)Axis-1 of the American Psychiatric Association [17]. The instrument has been reported to have good reliability [18]. It was used to confirm the diagnosis of schizophrenia in the study population. The interviewers were trained in the use of the instruments by a certified trainer.
2. The Brief Psychiatric Rating Scale
  The Brief Psychiatric Rating Scale (BPRS) is an 18 item clinician-rated instrument designed to measure severity of psychopathology. The BPRS includes items that address somatic concern, anxiety, emotional withdrawal, conceptual disorganization, guilt feelings, tension, mannerisms and posturing, grandiosity, depressive mood, hostility, suspiciousness, hallucinatory behaviours, motor retardation uncooperativeness, unusual thought content, blunted affect, excitement, and disorientation.
  Thus, the items on the BPRS focus on symptoms that are common in patients with psychotic disorders, including schizophrenia. The items cover the broad range of symptoms that are commonly seen in psychiatry [19]. The BPRS provides a continuous total score that is most often used to assess the effectiveness of treatment interventions. The BPRS has shown good reliability and validity and it has been extensively used in Nigeria [19] The inter-rater reliability was 0.75.
3. Socio-demographic Questionnaire
  A questionnaire was used to capture important socio-demographic and socio-economic data, such as age, occupation, tribe, address, employment status, length of relationship, monthly income of participants, and duration of illness.
4. Intimate Partner Violence questionnaire.
  The 20 item purposely designed questionnaire used in the study was based on an extensive focus group discussion on the items that were extracted from World Health Organisation Violence against women (WHOVAW) multi-country study questionnaire on intimate partner violence [6]. The questionnaire was limited to 20 items of measure for ease of administration and quick assessment and was interviewer administered(by residents psychiatrists). The questionnaire covered the 3 basic forms of IPV which were physical abuse, verbal abuse and sexual assault. Participants were given the choice of either to answer a Yes or No. The questions spread across these 3 domains. A)Physical abuse B) Verbal Abuse (C) sexual Assault. The internal consistency was high (α =0.80).

### Data analysis

Socio-demographic and socio-economic variables were presented in frequencies and percentages. Chi-squares were used to explore association of categorical socio-demographic variables with IPV. Independent *t*-test was used to explore differences in means of variables with IPV and pattern of IPV with mean scores on the BPRS. Effect sizes were calculated for significant associations.

## Results

Seventy –seven persons (77) completed the questionnaire out of ninety –one (91) that were recruited for the survey. Eleven persons refused further participation while three (3) persons did not adequately fill the questionnaire items such as age. The participation rate was 85 %.

1. Socio -demographic/Socio-economic variables (Table 1)

**Table 1:** Socio-demographics/economics variable (*N* = 77)

Variables	Frequency	Percentage	Mean ± SD
Age (years)			
21–41	54	70.0	38.3 ± 2,8
42–62	19	25.0	
63–83	4	5.0	
Tribe			
Efik/Ibibio	38	49.0	
Ibos	26	34.0	
Yoruba	12	16.0	
Hausas	1	1.0	
Religion			
Christianity	58	75.0	
Islam	13	17.0	
Traditional	6	8.0	
Employment status			
Employed	10	13.0	
Self-employed	42	55.0	
Unemployed	25	32.0	
Education(YEARS)			
Nil	10	13.0	
1–6(primary)	13	17.0	
7–12(secondary)	35	45.0	
>12(tertiary)	19	25.0	
Length of intimate relationship(YEARS)			
≤10	41	53.2	
11–21	21	27.3	
22–32	11	14.3	
33–43	4	5.2	
Duration of illness(YEARS)			
≤10	55	71.0	
11–21	20	26.0	
22–32	2	3.0	
Medication adherence			
Regular	57	74.0	
Irregular/non compliant	20	26.0
  The mean age of the participants was 38.3 ± 2.8 years. Close to half, 49 % (*n* = 38) belonged to the Efik/Ibibio tribe, 75 % (*n* = 58) were Christians. A total of 35 (45 %) participants had up to 12 years of education. More than half (53.2 %, *n* = 41) had been in relationship with their partners//husband less than or close to 10 years. Majority (71 %, *n* = 55) had been diagnosed with schizophrenia within the last 10 years. Fifty-seven participants (74 %) reported full compliance with their medications. Close to a third (32 % *n* = 25) were unemployed (Table 1). Among the employed, mean income per annum was ₦16000 ± 3000 (≈ $ 100) which was a little lower than the official minimum wage of ₦18000 ($ 107).
2. Pattern of intimate pattern of violence
  A total of 58(75 %) participants reported at least one incidence of intimate partner violence out of the 77 participants. The most frequently reported form of Intimate Partner was verbal abuse (73 % *n* = 56). A total number of 41 participants (53 %) reported a history of physical abuse. Sexual assault was reported by 19 participants (25 %). Among those who reported physical abuse, seventeen participants (41 %) had injuries as a result of the physical abuse. Close to half (49 % *n* = 38) of the participants reported incidence of physical and verbal abuse. All the 19 (25 %) participants that reported sexual assault also reported verbal abuse. Eight (10 %) participants that reported physical and sexual assault also reported verbal abuse.
3. Intimate Partner Violence across Socio-demographic variables (Table 2)

**Table 2:** Intimate partner violence across socio-demographic variable

Variables	YES	NO	Test statistic	DF
Verbal Abuse				
Age	M = 35.30(9.00))	M = 43.70(10.80)	t = −3.43* (95 % cl −13.19 - 3.50)	75
Education	M = 12.82(1.01)	M = 12.80 (0.81)	t = 0.05 (95 % cl – 0.48- 0.50)	75
Duration of illness	M = 7.14(5.30)	M = 10.15(8.26)	t = −1.83 (95 % CL −6.29-0.26)	75
Length of relationship	M = 12.00(8.56)	M = 16.52(11.38)	t = −1.83 (95 % CL −9.46-0.41)	75
Physical Abuse				
Age	M = 35.67(8.71)	M = 39.68(11.26)	t = −1.75 (95 % CL −8.56-0.56)	75
Education	M = 12.90(1.01)	M = 12.76(0.90)	t = −0.54 (95 % CL −0.32-0.60)	75
Duration of illness	M = 6.61(4.84)	M = 9.37(7.35)	T = 1.92 (95 % CL-5.61, 0.10)	75
Length of relationship years	M =12.92	M = 13.40(10.70)	T = −0.22 (95 % CL-4.87- 3.93)	75
Sexual Abuse				
Age	M = 32.68(9.55)	M = 39.20(9.90)	t = −2.51* (95 % CL −11.69-1.35)	75
Education(Years)	M = 12.74(0.87)	M = 12.84(0.98)	t = −0.84 (95 % CL −4.83-1.96)	75
Duration of illness(Years)	M = 6.83(6.00)	M = 8.26(6.37)	t = −0.84 (95 % CL −4.82,-1.95)	75
Length of relationship (years)	M = 8.80(8.31)	M = 14.62(9.46)	t = −2.39* (95 % CL −10.70-9.72)	75

*Significance at *p* < 0.050
  Results of the independent *t*-test showed that there were significant mean difference in ages of those who reported history of Verbal abuse (M = 35.30, SD = 9.00) compared to those who did not report such cases (M = 43.70, SD = 10.30) at *p* < 0.05 at (t = −3.38, df = 75, 95%CI mean difference −13.19-3.50, Cohen’s d = 0.87, *r* = 0.39). Also, mean differences of those who reported sexual assault compared to those who had no such history was significant at *p* < 0.05 (t = 2.51 df = 75, 95 % CL −11.69-1.35 Cohen’s d = 0.67, *r* =0.31). Verbal and sexual assault was commoner among those who were younger in age. Sexual Assault was significantly associated with length of intimate relationship at *p* < 0.05 (t = −2.39, df = 75, 95 % CL −10.70-9.72 Cohen’s d = 0.67, *r* =0.31). Sexual Assault was higher in those who had a lower mean length of relationship (Table 3). Pearson’s chi-square test was done to explore the relationship between employment and medication adherence with the three forms of IPV (Table 2).

**Table 3:** Intimate partner violence and psychopathology

Variable	BPRS M(SD)	T	df	95 % CL	P
IPV					
YES	28.71(8.56)	1.40	75	-1.57- 7.81	0.20
NO	25.58(8.24)				
Verbal Abuse					
YES	28.74(8.73)				
		1.30	;	-1.53-7.42	0.21
NO	25.84(7.84)				
Physical Abuse					
Yes	29.84(8.24)				
		1.51	;	-0.96-6.96	0.14
No	26.84(8.71)				
Sexual assault					
Yes	30.70 (9.40)				
		1.62	;	-0.85-8.14	0.11
No	27.40(8.11)				
Verbal and Physical					
YES	29.50(8.46)				
		1.51		-0.96-6.94	0.66
NO	26.47(8.47)				
Physical,Sexual, Verbal					
Yes	37.50(5.34)	3.61			
				-4.78-16.59	
No	26.81(8.14)				0.00*

*Significance at *p* < 0.05
  Employment status was significantly associated with Verbal Abuse, *x*^2^(1, *N* = 77) = 17, *p* = 0.01, those who were unemployed significantly reported higher number of cases of verbal abuse (92.3 %) compared to those with employment (62.7 %). Verbal abuse was significantly associated with Medication Adherence *X*^2^(1, *N* = 77) = 5.05, *p* = 0.03. Verbal abuse was commoner among those who did not comply with medication (94.7 %) compared to those who complied with their medication (69.1 %).
4. Intimate Partner Violence and Psychopathology
  The mean scores on the BPRS are as shown in Table 3. Across all forms of IPV, victims of IPV had higher mean scores on the BPRS. Significant mean differences on the BPRS were seen in victims who reported all three forms of IPV (M = 37.50, SD = 5.34) compared to those without such history at (M = 26.81, SD = 8.14) at *p* = 0.01 (t =3.61, df = 75, 95 % CI −4.78-16.59, Cohen’s d = 1.60, *r* =0.61). Women with schizophrenia who suffer intimate partner violence reported higher psychopathology on the BPRS. Interestingly, all those who were victims of sexual and physical violence also reported verbal abuse.

## Discussion

Our study surveyed the pattern of prevalence of various kinds of intimate partner violence among the population of female patients with schizophrenia attending the clinic. As observed in our study, there was a high prevalence of intimate partner violence (75 %) reported by participants in our sample. The rate was higher than rates reported in previous studies on domestic violence among a similar population involving psychiatric patients [16, 20]. Our findings show that women with schizophrenia in intimate relationship suffer a high degree of IPV The high rate may be partly due to the nature of the disorder which makes them vulnerable to such violence [12, 13]. The high rate was reported despite the socio-cultural barriers to disclosure in Nigeria [21]. In comparison, the rate in our study population was higher than that reported by Olayanju et al. [11].

The most prevalent form of IPV in our study was Verbal abuse. Close to three-quarter of our respondents (73 %) reported acts of abuses, insults and critical comments by intimate partners. Regrettably, verbal abuse is a major form of violence that is not often explored in surveys of intimate partner violence but constitutes a very frequent and distressing form of IPV. Verbal abuse extols a high emotional toil that negatively affects patients with schizophrenia. The importance of this form of IPV lies in the influence it has on victims because it represents attempt to control, coerce and intimidate [6, 22]. It can also be a prelude for physical violence [6]. Importantly, it has been shown that excessive critical comments impact negatively on the rate of relapse in patients with schizophrenia [23]. Sadly, not often do mental health professionals ask about this form of violence in routine clinical visits [24].

Physical abuse was reported by more than 50 % of our respondents. Physical abuse involved various acts of beatings, hitting and physical punishments as reported by of the participants and a high percentage of these physical violence resulted in injuries to victims. The high degree of physical violence observed among women with schizophrenia may lead to further social deficits that impairs their capacity and ability to negotiate their way out of dangerous and potentially violent encounters with partners [25]. The very nature of the disorder gives rise to cognitive and emotional distortions that may also make them vulnerable to violence. About a quarter (24.1 % *n* = 19)) of the respondent reported incidences of sexual assault with intimate partners. Sexual assault is a severe form of IPV and women with schizophrenia are at increased risk [26]. Understandably, the lower rate may be due to the cultural beliefs which usually places undue stigmatisation towards women and limits the reporting of such cases [21]. It is noteworthy that those who reported a history of sexual and physical abuse also reported verbal abuse, this seems to suggest that women who report sexual assault and physical abuse may suffer from all other forms of IPV and precipitate serious fatal consequences [4, 27]. Unfortunately, it is rare for victims with the disorder to disclose such issues and hence often go undetected by clinicians involved in their care.

### Socio-demographics variables associated with IPV

In our study, we explored socio-demographic and clinical variables that were significantly associated with various forms of intimate violence. Age was the only socio-demographic variable significantly associated with two forms of intimate partner violence, verbal abuse and sexual assault. Length of relationship with partner was significantly associated with sexual assault. Those with lower duration of intimate relationship were more likely to report sexual assault. The result does suggest that relationships in which sexual assault are experienced usually are short-lived. There is a higher likelihood for IPV to be reported by those in the younger age group similar to the results of previous studies and in the general population [11, 25]. This may be due to adjustment difficulties and sense of belonging that the new status of such relationships brings. Theorists have postulated that younger women tend to be more materially dependent on their partners giving higher risk for IPV especially in patients with severe mental illness [24]. We found that Verbal abuse was significantly commoner in those who were unemployed and who were not compliant with medication. Unemployment brings strain in intimate relationships even among healthy individuals, for women with schizophrenia, being unemployed may be due to the deficit caused by the disorder apart from the socio-economic situation. It is quite important for mental health professionals to make specific enquiries in women with schizophrenia who are unemployed. This will help in instituting a comprehensive management strategy that would include social rehabilitation to help them gain some form of employment apart from the usual routine encouragement of medication adherence at clinics. However, there were no socio-demographic or clinical variable that were associated with physical abuse. It was interesting to find that numbers of years of formal education and duration of illness were not significantly associated with any form of IPV. Social interventions involving employment generation may help in reducing the burden of IPV in women with schizophrenia.

### IPV and association with psychopathology

We further explored the association of psychopathology (Using BPRS) with various forms of IPV. The findings reveal than those who reported all forms of IPV had higher mean score on the BPRS in comparison to those who did not report. Notably, as several studies have shown, psychiatric symptoms worsen with victimisation, leads to increased rate of psychiatric visitations and risk of suicide [6, 12, 26, 27]. The findings in our study show that all who reported the 3 forms of IPV were significantly associated with higher psychopathology.

Interestingly, among the various forms of IPV, those who reported sexual assault were the fewest but they had a very high mean score on the BPRS. Sexual assault represents a very severe form of IPV as our study suggests. All victims who reported sexual abuse and physical abuse, also reported verbal abuse They significantly had higher psychopathology than those without IPV. Our data seems to suggest that all who experienced sexual assault and physical abuse will also experience verbal abuse. Those who experienced all forms of IPV are prone to higher psychopathology in addition to the negative consequence on the physical health such as injuries, gynaecological problems, depression of immunity and possibly untimely death apart from the psychopathological burden [6]. It also has been reported to affect maternal responsibilities to the child in Nigeria [28]. This form of IPV may complicate treatment approaches and increase relapses among women with schizophrenia.

### Study limitation

The report of the participant might be susceptible to information distortion which were not independently verified. Further, though efforts were made to recruit participants without cognitive disorders clinically, this cannot be totally excluded as schizophrenia is associated with varied cognitive problems which may distort their reports. The cross-sectional study cannot be used to generalise and explore all the other factors that may be associated with psychopathology in women with schizophrenia. Much larger pool of patients and longitudinal surveys will be needed. The study criteria excluded relationships that experienced violence or dating relationships or any other relationships that ended more than a year ago and these group of women were not captured in our results thus limiting the generalisation of our results.. The small size sample of our study and the limitation to a geographical region cannot be used to predict the pattern of results for entire population of female patients with schizophrenia in Nigeria.

## Conclusion

The study highlighted the high rate of various forms of IPV among women with schizophrenia. Sexual assault and physical abuse are associated with higher score on the psychopathology scale.. The high prevalence of IPV among this group of patients creates a need for mental health professionals to identify potential victims by making routine enquiries about IPV among women with schizophrenia despite the difficulties in obtaining such information in Nigeria. Making direct and indirect enquiries about IPV will help clinicians to detect IPV and in planning holistic therapy that encompasses social interventions in helping women with schizophrenia.

## Abbreviations

BPRS, brief psychiatric rating scale; IPV, intimate partner violence.
